# Statins increase the risk of herpes zoster: A propensity score-matched analysis

**DOI:** 10.1371/journal.pone.0198263

**Published:** 2018-06-14

**Authors:** Min-Chul Kim, Sung-Cheol Yun, Sang-Oh Lee, Sang-Ho Choi, Yang Soo Kim, Jun Hee Woo, Sung-Han Kim

**Affiliations:** 1 Division of Infectious Diseases, Department of Internal Medicine, Chung-Ang University Hospital, Seoul, Republic of Korea; 2 Clinical Epidemiology and Biostatistics, Asan Medical Center, University of Ulsan College of Medicine, Seoul, Republic of Korea; 3 Departments of Infectious Diseases, Asan Medical Center, University of Ulsan College of Medicine, Seoul, Republic of Korea; University of British Columbia, CANADA

## Abstract

**Objectives:**

Statins, which are lipid-lowering agents, have anti-inflammatory and immunomodulatory properties that may affect the occurrence of various infectious diseases. We assessed whether statins increase the risk of herpes zoster (HZ) with propensity score-matching.

**Methods:**

The study was based on the National Health Insurance database and its subset database of the “medical check-up” population of South Korea. These cohorts consist of about one million and 570,000 people, respectively, representative of the entire population of South Korea. We identified 103,930 statin users and 430,685 non-statin users. After propensity score-matching, 25,726 statin users and the same number of non-statin users were finally analyzed. The development of HZ was monitored in these matched pairs over the 11 years from 2003 to 2013.

**Results:**

Statin users had a significantly higher risk of HZ than non-statin users: hazard ratio (HR) 1.25 (95% CI, 1.15 to 1.37) (*p* < .0001). The risk of HZ associated with statins was especially high in the elderly: HR 1.39 (95% CI, 1.12 to 1.73) in the over 70-year-olds (*p* = 0.003) and HR 1.18 (95% CI, 1.00 to 1.39) in the 60-to-69-year-olds (*p* = 0.056). Furthermore, there was a significant *p* for trend in terms of cumulative dose effect between the risk of HZ and the duration of statin use (*p* < .0001).

**Conclusions:**

These epidemiologic findings provide strong evidence for an association between HZ and statin use, and suggest that unnecessary statins should be avoided.

## Introduction

Inhibitors of 3-hydroxy-3-methylglutaryl coenzyme A (HMG-CoA) reductase, commonly known as statins, are lipid-lowering agents, which have been widely prescribed for primary and secondary prevention of cardiovascular diseases [1]. In addition to their effect on cholesterol metabolism, statins have anti-inflammatory and immunomodulatory actions that may have various effects on infectious diseases.

Herpes zoster (HZ), which is caused by reactivation of latent varicella zoster virus (VZV), afflicts about 30% of the global population overall and is accompanied by substantial morbidity [2]. The risk of HZ increases with age and diverse medical illnesses, by which VZV-specific T-cell immunity has waned [3]. Identifying the risk factors for HZ would have significant implications for public health, since some cases of HZ might be potentially prevented by correction of the modifiable risk factors.

Previous studies have reported that statin use is associated with increased risk of HZ [4–7]. However, the association has remained uncertain because confounding factors may not have been adequately controlled. In the present work, we adjusted comprehensively for confounding factors, and assessed the risk of HZ associated with statin use in a large population by propensity score matching.

## Methods

### Database source and study population

We used the database of the National Health Insurance (NHI) of South Korea and its subset database of the “medical check-up” population [8, 9]. The NHI is a nationwide healthcare system covering the entire Korean population, and these databases have been maintained by the National Health Insurance Service (NHIS) since 2002. The NHI cohort consists of about one million individuals and the “medical check-up” database comprised approximately 570,000 people who received medical check-ups. These databases provide comprehensive social and medical information about the population including prescription records. They are representative of the total population of South Korea of all ages and regions. The study protocol was approved by the Institutional Review Board of Asan Medical Center (S2015-0982).

### Identification of patients with herpes zoster, statin users, and confounding factors

We identified patients diagnosed with HZ using the relevant diagnostic codes of *the International Classification of Diseases*, *Tenth Revision* (ICD-10). The cases of HZ consisted of VZV meningitis (B020), VZV encephalitis (B021), zoster in other divisions of the trigeminal nerve (B022), zoster ophthalmicus (B023), and other types of zoster (B027, B028, or B029). Clinical characteristics that could be confounding factors for HZ and statin usage were searched using the ICD-10 code or medical records, as previously described [8, 9]. They included the following: age, gender, economic class, hypertension, diabetes, dyslipidemia, ischemic heart disease, transient ischemic attack, heart failure, atrial fibrillation/flutter, valvular heart disease, carotid stenosis, peripheral vascular disease, chronic renal disease, chronic liver disease, chronic pulmonary disease, rheumatoid disease, inflammatory bowel disease, malignancy, transplantation recipients of solid organs or hematopoietic stem cell, human immunodeficiency virus (HIV) infection, and depression.

Statin users were defined as individuals who had ever been prescribed any one of the 7 statins (simvastatin, atorvastatin, rosuvastatin, lovastatin, fluvastatin, pravastatin, and pitavastatin) available in South Korea. Non-statin users were defined as individuals who had never been prescribed statins. As was done in previous studies [4–6], we defined statin users as subjects who received prescription for statins continuously for at least 2 months, because we assumed that the duration of statin use could affect VZV reactivation. If HZ was developed within 2 months from the start of statins, the case was excluded as an event not to be associated with the statins. When HZ occurred in a statin user after he or she had stopped taking statins, it was regarded as statin-associated if it occurred within 2 months of stopping. If a statin user did not develop HZ within two months of stopping, the subject was censored, since the HZ which happened two months later from cessation of the drugs could not be asserted as a statin-associated event. Individuals who died or emigrated from South Korea were also censored (S1 Fig).

### Study design and outcomes

**Fig 1:**
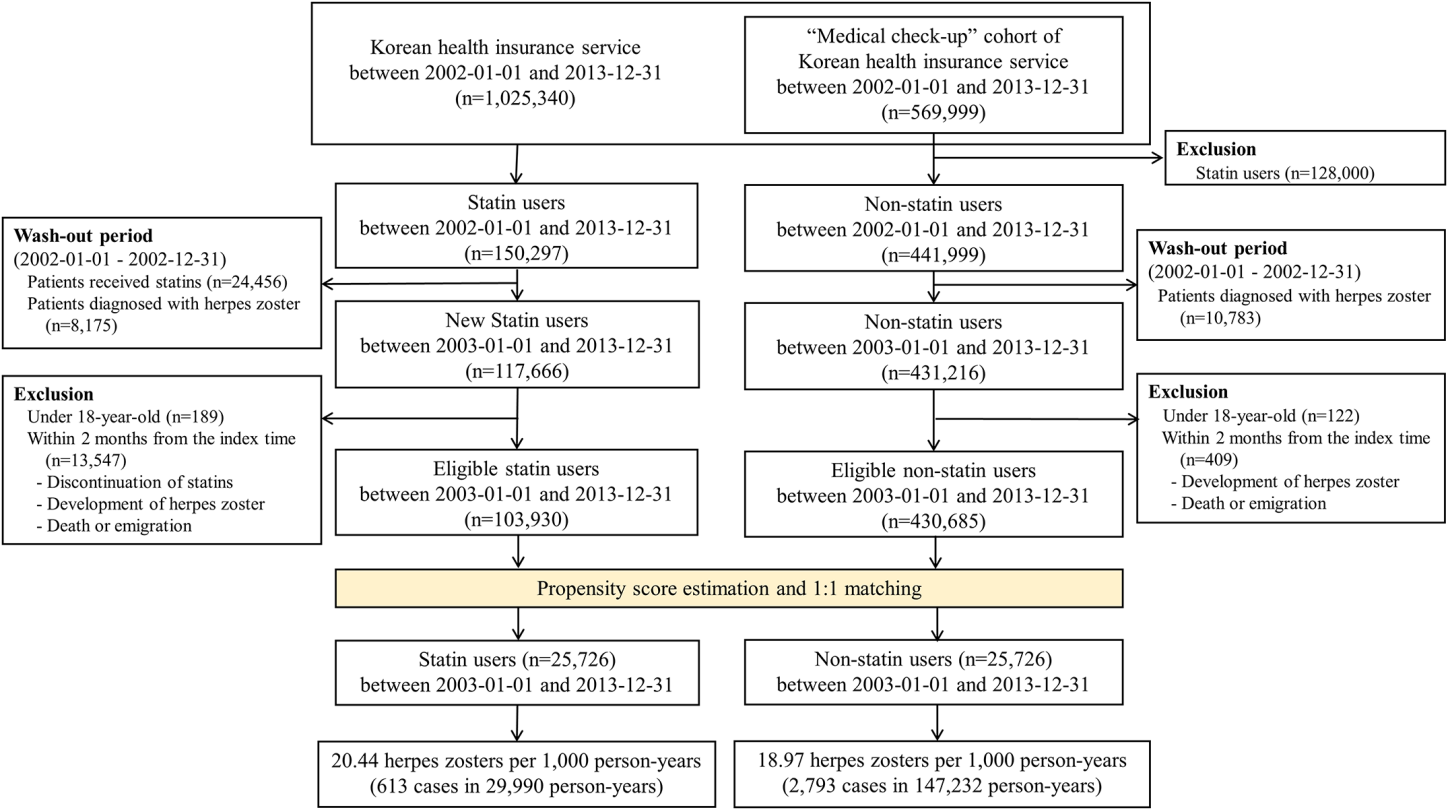
Flow diagram of the study. Flow diagram giving details of the enrolled individuals, and the procedure for propensity score matching.

We conducted a propensity score-matched analysis to evaluate the impact of statins on the risk of HZ. A total of 150,297 individuals who had used statins were identified among the 1,025,340 individuals in the NHI cohort (Fig 1). To be sure to identify new statin users and the first-ever episodes of HZ, we excluded individuals with prescription records of statins and with histories of HZ in the first year of the observation period, which we designated as a wash-out period. In this way we excluded 24,456 previous statin users and 8,175 patients who had a past histories of HZ. In addition, 189 new statin users who were < 18 years old were excluded. 13,547 individuals who discontinued statins, or developed HZ, or died or emigrated within 2 months from the index time, were also excluded. Finally, a total of 103,930 were enrolled in the statin group.

We used the “medical check-up” cohort to identify non-statin users to be matched with the statin users because of the need to determine an index time when the non-statin users were enrolled so that their propensity scores were compared with those of statin users in the relevant year. From the 569,999 individuals in “medical check-up” cohort, we excluded 128,000 who had used statins, leaving 441,999 non-statin users. A further 10,783 individuals with a past history of HZ in the wash-out period were excluded, together with 122 persons of < 18 years. 409 subjects who developed HZ or who died or emigrate within 2 months from the index time were excluded, to balance the excluded statin users within the corresponding periods. Thus, a total of 430,685 non-statin users were enrolled in the control group.

After propensity score-matching, 25,726 statin users and the same number of propensity score-matched non-statin users were monitored for the development of HZ over the 11 years from 2003 to 2013 (Fig 1). A schematic diagram of the study is shown in S1 Fig. We also performed subgroup analyses according to age and duration of statin use.

### Statistical analysis

Categorical data were compared with chi-square tests, and continuous variables by unpaired Student’s *t* tests. Differences between the statin users and the non-statin users in clinical characteristics were assessed by Cox proportional hazards analyses. Propensity scores were estimated without regard to the outcome variables, using multiple logistic-regression analysis. All covariates were included in the full non-parsimonious model for statin usage (Table 1) [8, 9]. The propensity score-matched pairs were created by matching the statin users and the non-statin users using calipers of width equal to 0.2 of the standard deviation of the logit of the propensity score. We employed the standardized difference (SD) to check for differences in baseline characteristics. For categorical variable, we used a multivariate Mahalanobis distance method to generalize the standardized difference metric. It has been suggested that a SD of < 10% probably denotes a negligible imbalance. The risk of HZ was compared using Cox regression models with robust standard errors. The proportional hazards assumption was confirmed by examining log (-log [survival]) curves and by testing partial (Schoenfeld) residuals, and no relevant violations were found. In addition, incidence rates per 1,000 person-years and exact Poisson confidence intervals (CI) were calculated. The cumulative incidence of HZ was constructed as Kaplan–Meier estimates according to the usage of statins. All reported *p*-values are two sided, and *p*-values <0.05 were considered statistically significant. Data manipulation and statistical analyses were conducted using SAS^®^ Version 9.2 (SAS Institute Inc., Cary, NC).

**Table 1:** Baseline clinical characteristics of the cohort.

	Total cohort population			Propensity-score matched population			
	Statin user (n = 103,930)	Non-statin user (n = 430,685)	*P*-value	Statin user (n = 25,726)	Non-statin user (n = 25,726)	SD (%)	*P*-value
**Age**, mean ± standard deviation (years)	56.8 ± 12.3	42.2 ± 14.2	< 0.001	52.6 ± 13.6	52.5 ± 13.7	0.59	0.51
18~30	1,690 (1.6)	111,138 (25.8)		1,460 (5.7)	1,380 (5.4)		
31~40	7,999 (7.7)	104,049 (24.2)		3,743 (14.6)	3,897 (15.2)		
41~50	22,302 (21.5)	108,318 (25.2)		6,616 (25.7)	6,881 (26.8)		
51~60	32,315 (31.1)	55,988 (13.0)		6,362 (24.7)	6,202 (24.1)		
61~70	24,982 (24.0)	33,928 (7.9)		4,846 (18.8)	4,747 (18.5)		
71~	14,642 (14.1)	17,264 (4.0)		2,699 (10.5)	2,619 (10.2)		
**Male gender**	47,816 (46.0)	221,709(51.5)	< 0.001	12,608 (49.0)	12,587 (48.9)	1.36	0.85
**Economic class**			< 0.001			2.07	0.24
Low (≤ 30%)	32,711 (31.5)	163,291 (37.9)		8,193 (31.8)	8,375 (32.6)		
Middle (30% ~ 80%)	38.929 (37.5)	179,818 (41.8)		10,026 (39.0)			
High (> 80%)	32,290 (31.1)	87,576 (20.3)		7,507 (29.2)	7,380 (28.7)		
**Hypertension**	61,401 (59.1)	39,577 (9.2)	< 0.001	11,084 (43.1)	11,029 (42.9)	0.43	0.62
**Diabetes**	37,621 (36.2)	22,477 (5.2)	< 0.001	7,839 (30.5)	8,114 (31.5)	-2.31	0.009
**Dyslipidemia**	99,468 (95.7)	22,254 (5.2)	< 0.001	21,267 (82.7)	21,284 (82.7)	-0.17	0.84
**Ischemic heart disease**	19,631 (18.9)	9,581 (2.2)	< 0.001	3,577 (13.9)	3,471 (13.5)	1.2	0.17
**Transient ischemic attack**	3,279 (3.2)	2,006 (0.5)	< 0.001	595 (2.3)	563 (2.2)	0.84	0.34
**Heart failure**	6,649 (6.4)	3,749 (0.9)	< 0.001	1,250 (4.9)	1,180 (4.6)	1.28	0.15
**Atrial fibrillation/flutter**	2,122 (2.0)	1,367 (0.3)	< 0.001	433 (1.7)	421 (1.6)	0.37	0.68
**Valvular heart disease**	987 (1.0)	692 (0.2)	< 0.001	211 (0.8)	222 (0.9)	-0.47	0.60
**Carotid stenosis**	687 (0.7)	71 (0.0)	< 0.001	54 (0.2)	44 (0.2)	0.89	0.31
**Peripheral vascular disease**	15,597 (15.0)	9,760 (2.3)	< 0.001	2,751 (10.7)	2,773 (10.8)	-0.28	0.75
**Chronic renal disease**	3,184 (3.1)	2,235 (0.5)	< 0.001	904 (3.5)	948 (3.7)	-0.92	0.30
**Chronic liver disease**	1,877 (1.8)	2,780 (0.7)	< 0.001	672 (2.6)	765 (3.0)	-2.19	0.013
**Chronic pulmonary disease**	29,389 (28.3)	68,705 (16.0)	< 0.001	7,028 (27.3)	7,365 (28.6)	-2.92	0.001
**Rheumatoid disease**	7,160 (6.9)	12,023 (2.8)	< 0.001	1,857 (7.22)	1,969 (7.7)	-1.66	0.060
**Inflammatory bowel disease**	377 (0.4)	1,264 (0.3)	< 0.001	137 (0.5)	144 (0.6)	-0.37	0.68
**Malignancy including solid cancer and hematologic malignancy**	4,717 (4.5)	7,981 (1.9)	< 0.001	1,292 (5.0)	1,463 (5.7)	-2.95	0.001
**Recipients of solid organ transplantation or hematopoietic stem cell transplantation**	115 (0.1)	160 (0.0)	< 0.001	30 (0.1)	22 (0.1)	-0.46	0.27
**Human immunodeficiency virus infection**	11 (0.0)	40 (0.0)	0.70	4 (0.0)	2 (0.0)	0.72	0.41
**Depression**	1,437 (1.4)	2,197 (0.5)	< 0.001	308 (1.2)	354 (1.4)	-1.59	0.072

Abbreviations: SD, Standardized differences;

NOTE. Data are no. (%) of positive patients unless indicated otherwise.

## Results

### The study population

The baseline clinical characteristics of the statin users and non-statin users are shown in Table 1. Female gender was more common in the statin users, and the statin users had a higher socioeconomic status. Risk factors for atherosclerosis such as old age, hypertension, diabetes, dyslipidemia, chronic renal disease, chronic pulmonary disease, rheumatoid disease, malignancy were more common in the statin user group. Statin users also had more cardiovascular diseases such as ischemic heart disease, transient ischemic attack, heart failure, atrial fibrillation/flutter, valvular heart disease, carotid stenosis, peripheral vascular disease than non-statin users. After propensity score-matching, the SD for each adjusted variable was < 10% (Table 1).

### Risk of herpes zoster according to statin use

The incidence of HZ was higher in the statin users than in the non-statin users (Table 2). In the total population, the number of first-ever diagnosed HZ cases among the statin users was 3,172 in 142,883 person-years (22.20 per 1,000 person-years; 95% CI, 21.43 to 22.98), and, in the non-statin users, 33,787 in 2,875,489 person-years (11.75 per 1,000 person-years; 95% CI, 11.63 to 11.88). The hazard ratio (HR) of HZ was 1.53 (95% CI, 1.47 to 1.59) (*p* < .0001).

**Table 2:** Risks of herpes zoster in statin users and non-statin users.

	Statin users		Non-statin users		Hazard ratio (95% CI)	*P*-value
	Number of herpes zoster	Incidence per 1,000 person-years	Number of herpes zoster	Incidence per 1,000 person-years		
**Crude analysis**^a^	3,172	22.20 (21.43–22.98)	33,787	11.75 (11.63–11.88)	1.53 (1.47–1.59)	< 0.0001
**Propensity-score matching analysis**	613	20.44 (18.82–22.06)	2,793	18.97 (18.26–19.67)	1.25 (1.15–1.37)	< 0.0001
**Age**, years						
~39	23	7.10 (4.20–10.00)	324	11.10 (9.89–12.31)	0.81 (0.52–1.28)	0.37^b^
40~59	95	14.27 (11.40–17.14)	689	16.92 (15.66–18.19)	1.06 (0.86–1.32)	0.57^b^
50~59	180	21.49 (18.35–24.63)	805	22.25 (20.71–23.78)	1.06 (0.90–1.26)	0.48^b^
60~69	193	25.97 (22.31–29.63)	678	24.27 (22.44–26.10)	1.18 (1.00–1.39)	0.056^b^
70~	122	28.49 (23.44–33.55)	297	22.42 (19.87–24.97)	1.39 (1.12–1.73)	0.003^b^

Abbreviations: CI, confidential intervals

^a^ The crude analysis was performed in the total cohort population.

^b^ Subgroup analysis according to age group adjusted age and gender.

**Fig 2:**
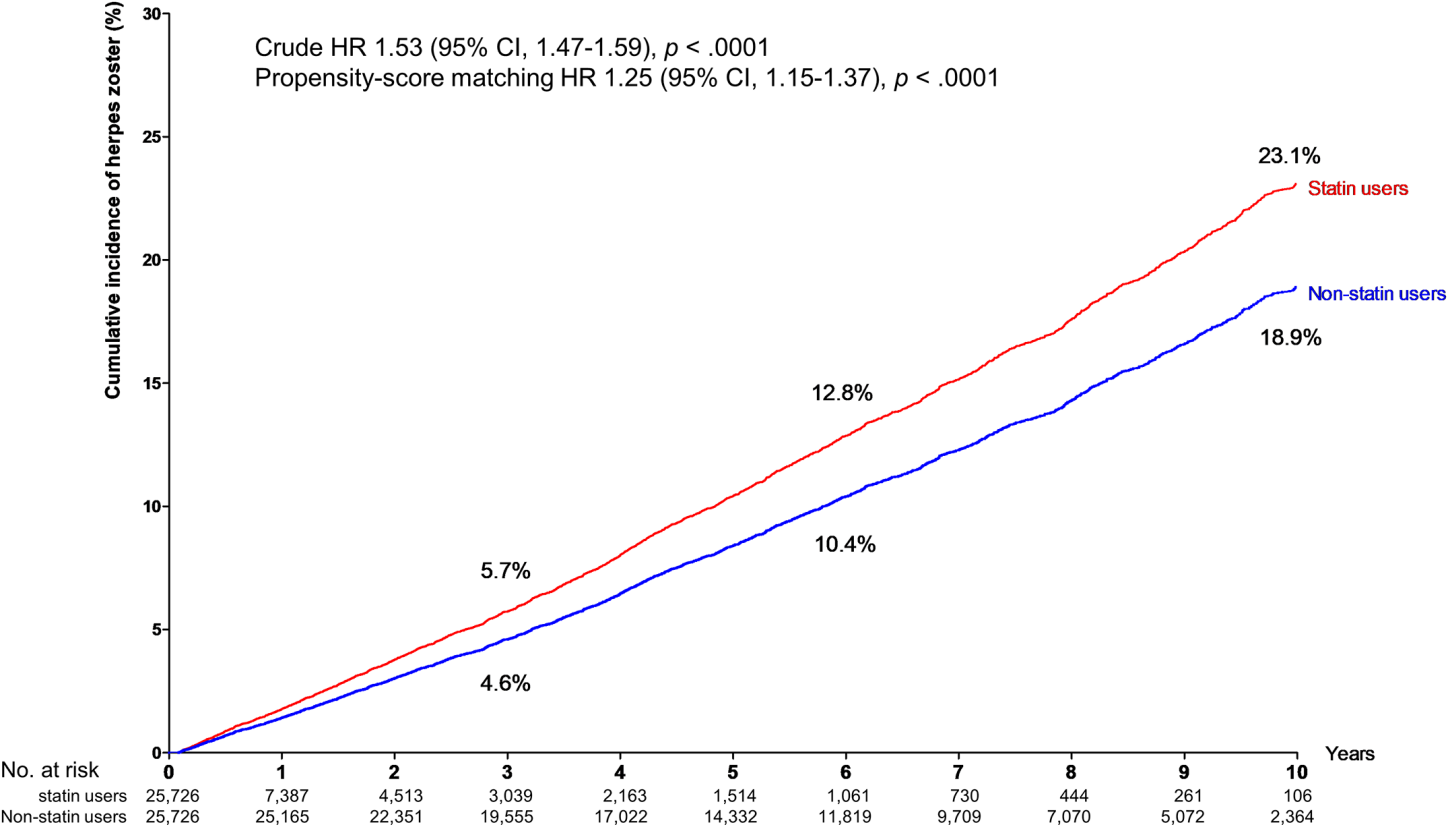
Cumulative incidences of herpes zoster (%) according to the use of statins. Kaplan–Meier curves showing cumulative incidences (%) of individuals suffering from herpes zoster. Red line indicating herpes zoster in statin users. Blue line depicting herpes zoster in non-statin users. The difference between statin users and non-statin users was significant (*p* < .0001 by the log rank test).

In the propensity score-matched analysis, the number of first-ever diagnosed HZ cases was 613 in 29,990 person-years (20.44 per 1,000 person-years; 95% CI, 18.82 to 22.06) in the statin users, and 2,793 in 147,232 person-years (18.97 per 1,000 person-years; 95% CI, 18.26 to 19.67) in the non-statin users (HZ 1.25 (95% CI, 1.15 to 1.37) (*p* < .0001). As shown in Fig 2, there was a significant difference between the cumulative incidences of HZ between the two groups (*p* < .0001). Interestingly, older statin users had significantly higher risks of HZ than younger statin users even after adjusting age and gender between the different age groups (Table 2): HR 1.39 (95% CI, 1.12 to 1.73) in the over 70s (*p* = 0.003), and HR 1.18 (95% CI, 1.00 to 1.39) in the 60-to-69 year-olds (*p* = 0.056). However, we found no appreciable differences of risks for HZ in the subgroups below 60.

### Risk of herpes zoster as a function of duration of statin use

The risk of HZ is shown as a function of duration of statin use in Table 3. The risk increased with length of statin exposure: relative to those who used statins for less than 6 months, the odds ratios (ORs) were 2.87 (95% CI, 2.17 to 3.81) in 6-to-12 month users, 3.95 (95% CI, 2.87 to 5.43) in 12-to-18 month users, 7.40 (95% CI, 5.46 to 10.04) in 18-to-24 month users, and 8.06 (95% CI, 6.51 to 9.97) in users for over 24 months (Table 3). There was a significant *p* for trend in terms of the cumulative dose effect between the risk of HZ and duration of statin exposure (*p* < .0001). This outcome was unchanged after adjustment of age and gender (*p* < .0001) (Table 3).

**Table 3:** Dose response relationship between duration of statin use and incidence of herpes zoster.

Duration of statin use	Number of individuals (%)	Number of herpes zoster (%)	Odds ratio (95% CI)	Adjusted^a^ odds ratio (95% CI)
**Total**	25,726 (100)	613 (100)		
~ 6 months	14,896 (57.9)	124 (0.83)	Reference	Reference
6 ~ 12 months	3,441 (13.4)	81 (2.35)	2.87 (2.17–3.81)	2.57 (1.94–3.41)
12 ~ 18 months	1,746 (6.8)	56 (3.21)	3.95 (2.87–5.43)	3.38 (2.45–4.66)
18 ~ 24 months	1,128 (4.4)	66 (5.85)	7.40 (5.46–10.04)	6.29 (4.62–8.56)
24 months ~	4,515 (17.6)	286 (6.33)	8.06 (6.51–9.97)	6.57 (5.29–8.16)
***p* for trend**			< 0.0001	< 0.0001

Abbreviations: CI, confidential intervals

^a^ Age and gender were adjusted for calculating odds ratio.

## Discussion

We have demonstrated that statins significantly increased the risk of HZ in a large population cohort even after rigorously adjusting possible confounding factors. In a propensity score-matched analysis, statins raised the risk of HZ by 25%. The risk was especially elevated (by 39%) in statin users over 70. Furthermore, there was a significant cumulative dose effect between the risk of HZ and the duration of statin exposure. Therefore, our findings provide important evidence for an increased risk of HZ associated with statins. This suggests that unnecessary statins should not be prescribed.

Although previous epidemiologic studies [4–7] have reported similarly elevated risks of HZ after statin use, they had important limitations. Antoniou et al [4] found that individuals exposed to statin had a 13% increased risk of HZ compared with non-statin user in an Ontario cohort. However, their study was confined to individuals ≥ 66 years old and they did not detect any dose-responsiveness of the risk of HZ, probably due to the limited number of patients who increased their statin dose. From the Taiwan population, increased risks of HZ related to statin use were reported by Chung et al (28%) [5] and by Chen et al (21%) [6]. Both studies noted higher risks in female statin users and relatively young users. The latter finding contrasts with the results reported here with regard to vulnerable age group. However, only a limited number of clinical characteristics of the populations were included in those studies, so unmeasured confounding factors in their studies such as dyslipidemia and ischemic heart disease may have contributed to this discrepancy. Matthews et al [7] found that statins raised the risk of HZ by 13% in the UK population; the risk was dose-dependent and was reduced by stopping the drugs. However, this study employed a case-control study design, matching GP practice, age, and gender. Therefore, the true association between HZ and statin use could not be evaluated due to inadequate control of confounding factors.

There have been several attempts to use statins as an adjuvant therapy in sepsis [10–14] and pneumonia [15–21] to control dysregulated immune responses of hosts. The postulated mechanisms of protectiveness of statins were lowering the proinflammatory responses of macrophages and neutrophils via various intracellular signaling pathways and cytokines, limiting endothelial cell activation, and inhibiting expression of major histocompatibility complex (MHC)-II genes [22, 23]. However, findings concerning the protectiveness of statins in these diseases have been conflicting, probably due to the complexity of the immunomodulatory actions of statins which do not target individual mediators, the diversity of the causative agents of the infectious diseases examined, and the heterogeneous study design [24–26]. Optimism regarding the protective effects of statins in infectious diseases should not be directly extended to the link between HZ and statins: HZ is a well-documented disease entity caused by a single viral pathogen (VZV) and the increased risk of HZ associated with statin use has been consistently observed in several epidemiologic studies [4–7]. Statins inhibit interferon-γ-induced MHC-II expression and prevent antigen presentation to CD4^+^ T cells [27]. Statins also enhance immunosuppressive activity of regulatory T cells [28]. Putting these together, statins could theoretically lead to reactivation of VZV. It is worth noting that statin use may be associated with decreased VZV-specific cell-medicated immune responses to vaccination against HZ. Irwin et al. showed that untreated major depression, which is a risk factor for development of HZ, reduced immune responses to vaccination against HZ, and antidepressant therapy was associated with normalization of the immune responses [29]. These findings suggest that risk factors for the development of HZ could also suppress immune responses to vaccination against HZ. In addition, statin usage is associated with a reduced efficacy of influenza vaccination [30–32]. Therefore, further studies to establish whether statins decrease immune responses to vaccination against HZ are required.

The immunosuppressive property of statins could lead to concern that they may suppress immune surveillance against tumor development. Statins can exhibit immune tolerance induced by regulatory T cells which suppress tumor-specific effector T-cell responses [28, 33]. However, there have been conflicting data suggest that statins promote [34, 35] or prevent [36, 37] cancers. The complexity of the association between statins and cancers might be attributable to the shared risk factors between statin-eligibility and cancers, and to the interplay of such confounding factors [38]. Until now, there was a lack of definite evidence about the association between statins and cancers [39]. However, given the worldwide usage of statins and its implications for infections and cancers, further experimental and epidemiologic studies are required [40].

Our study has several limitations. First, we could not include body mass index, smoking status, alcohol use and cholesterol level as confounding factors, as the required data were not available in the NHI database. Cholesterol level and the status of the apolipoprotein E epsilon 4 allele (APOE4), in particular, have been suggested to be relevant risk factors for HZ [41, 42]. These unmeasured confounding factors might have affected our results. Second, some may be concerned that this study used two different cohorts, the NHI cohort for identifying statin users and the medical check-up cohort for non-statin users, despite the need for the index time for propensity score-matching. The latter cohort could be biased towards individuals who pay more attention to medical problems and are more prosperous and healthier than those in the former cohort. Therefore, the risk of HZ associated with statins may have been overestimated. Third, questions might be raised about missing data for the cohorts. The data used are based on claims for medical expenses to the national health insurance system, a single insurer [43], so theoretically there should be no missing data since none of the covariates in the study included non-insurance practices. However, individuals may be censored if they die or emigrate. Finally, the validity of HZ diagnosis based on ICD codes might be challenged. However, these diagnostic codes have been validated and shown to have high sensitivities and positive predictive values (> 85%) for identifying new cases of HZ [44].

In conclusion, our epidemiologic findings provide strong evidence for an association between HZ and statin use. This suggests that one should avoid unnecessary use of statins.

## Supporting information

S1 Fig Schematic diagram of the study. (TIF)

## Abbreviations

CI: Confidence interval
HIV: human immunodeficiency virus
HR: hazard ratios
HZ: herpes zoster
ICD diagnostic code: International Classification of Diseases diagnostic code
NHI: National Health Insurance
SD: standardized difference
TIA: transient ischemic attack
VZV: varicella zoster virus
