# Supplementary figures and images for: Statins increase the risk of herpes zoster: A propensity score-matched analysis
Source: PLoS One. 2018 Jun 14;13(6):e0198263. doi: 10.1371/journal.pone.0198263 (PMC6001979; doi:10.1371/journal.pone.0198263)

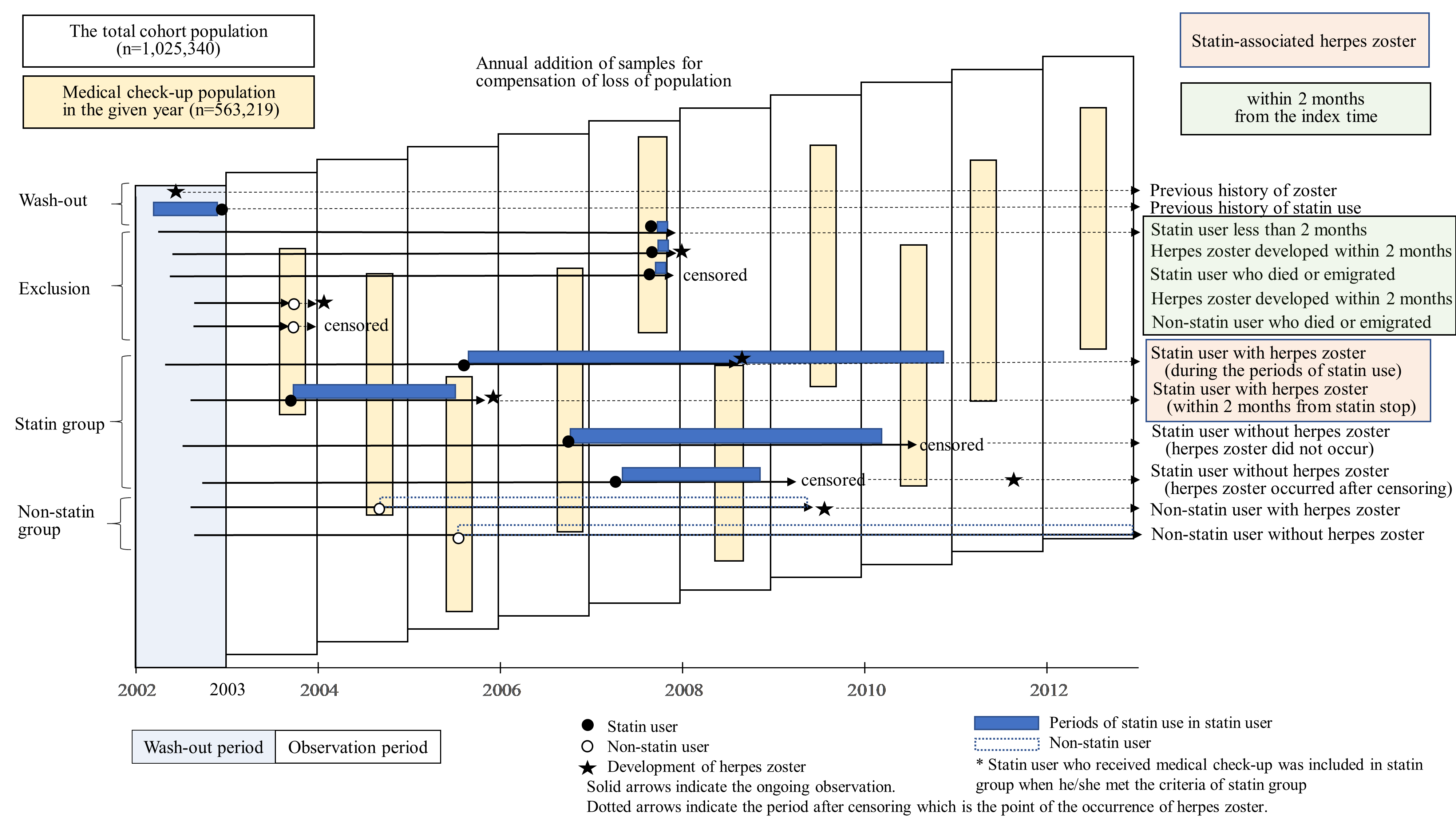

Supplement: S1 Fig — (TIF) [file pone.0198263.s001.tif]
